# Brain structural correlates of an impending initial major depressive episode

**DOI:** 10.1038/s41386-025-02075-6

**Published:** 2025-03-12

**Authors:** Anna Kraus, Katharina Dohm, Tiana Borgers, Janik Goltermann, Dominik Grotegerd, Alexandra Winter, Katharina Thiel, Kira Flinkenflügel, Navid Schürmeyer, Tim Hahn, Simon Langer, Tilo Kircher, Igor Nenadić, Benjamin Straube, Hamidreza Jamalabadi, Nina Alexander, Andreas Jansen, Frederike Stein, Katharina Brosch, Paula Usemann, Lea Teutenberg, Florian Thomas-Odenthal, Susanne Meinert, Udo Dannlowski

**Affiliations:** 1 Institute for Translational Psychiatry, University of Münster, Münster, Germany; 2 Department of Psychiatry and Psychotherapy, University of Marburg, Marburg, Germany; 3 Center for Mind, Brain and Behavior (CMBB), University of Marburg and Justus Liebig University Giessen, Giessen, Germany; 4 Core-Facility Brainimaging, Faculty of Medicine, University of Marburg, Marburg, Germany; 5 Institute for Translational Neuroscience, University of Münster, Münster, Germany

**Keywords:** Neuroscience, Depression

## Abstract

Neuroimaging research has yet to elucidate whether reported gray matter volume (GMV) alterations in major depressive disorder (MDD) exist already before the onset of the first episode. Recruitment of presently healthy individuals with a subsequent transition to MDD (converters) is extremely challenging but crucial to gain insights into neurobiological vulnerability. Hence, we compared converters to patients with MDD and sustained healthy controls (HC) to distinguish pre-existing neurobiological markers from those emerging later in the course of depression. Combining two clinical cohorts (*n* = 1709), voxel-based morphometry was utilized to analyze GMV of *n* = 45 converters, *n* = 748 patients with MDD, and *n* = 916 HC in a region-of-interest approach and exploratory whole-brain. By contrasting the subgroups and considering both remission state and reported recurrence at a 2-year clinical follow-up, we stepwise disentangled effects of (1) vulnerability, (2) the acute depressive state, and (3) an initial vs. a recurrent episode. Analyses revealed higher amygdala GMV in converters relative to HC (*p*_tfce-FWE_ = 0.037, *d* = 0.447) and patients (*p*_tfce-FWE_ = 0.005, *d* = 0.508), remaining significant when compared to remitted patients with imminent recurrence. Lower GMV in the dorsolateral prefrontal cortex (*p*_tfce-FWE_ < 0.001, *d* = 0.188) and insula (*p*_tfce-FWE_ = 0.010, *d* = 0.186) emerged in patients relative to HC but not to converters, driven by patients with acute MDD. By examining one of the largest available converter samples in psychiatric neuroimaging, this study allowed a first determination of neural markers for an impending initial depressive episode. Our findings suggest a temporary vulnerability, which in combination with other common risk factors might facilitate prediction and in turn improve prevention of depression.

## Introduction

Despite considerable effort, neuroimaging research has yet to unravel whether brain structural alterations in major depressive disorder (MDD) exist already before the onset of the initial depressive episode, indicating a neurobiological vulnerability. Meta-analyses of cross-sectional neuroimaging studies reported morphological changes in patients with MDD compared to healthy controls (HC) [1–3], particularly in areas of the frontolimbic circuity [4, 5]. While reduced gray matter volumes (GMV) are commonly observed in cortical regions [2, 3, 6, 7], findings in subcortical regions are more heterogeneous [1, 7, 8]. For example, GMV alterations of the amygdala vary across studies and may depend on disease stage, psychopharmacological intake, and familial risk for MDD [9–11]. Due to the study designs and samples examined, these studies provide very limited insight into the temporal occurrence and persistence of GMV changes in MDD: are these alterations pre-existing and represent a neural vulnerability for the future onset of the disorder or do they emerge later as a correlate of MDD developing in the course of the disorder? And further, are changes in GMV of persistent nature or do they follow the dynamic course of the disorder, e.g., emerging only during acute depressive episodes and disappearing in times of remission [12]?

Consequently, we lack a consistent neurobiological theory to explain the complex interplay between depression and brain structure. Available studies have shown less GMV in MDD related to the number of recurrent episodes, duration of illness [8, 13–16], and longitudinal as a function of relapse [17–19] or rehospitalization [20] in cortical and, with the exception of the amygdala, limbic structures. Supporting the assumption of a rather dynamic correlate, greater amygdala GMV has been reported in association with a current first depressive episode [21, 22] while longitudinal studies found support for state-like volume decrease in both cortical and subcortical regions driven by current depressive symptoms [17, 23, 24]. There is a crucial gap in our understanding of how GMV changes may serve as a marker for the future onset of MDD. To shed light on neurobiological vulnerability, we need to examine currently healthy individuals with a subsequent transition to MDD (converters) as this is indispensable to determine pre-existing changes prior to the onset of MDD (i.e., the onset of a first episode). We face a staggering lack of studies in converter samples, while the little available research was conducted mainly on children and adolescents. These studies found reduced GMV in frontal and temporal areas, insula, and rostral anterior cingulate cortex (rACC) to be associated with the future onset of MDD or rise in depressive symptoms [25, 26]. Two studies reported enhanced amygdala volumes [27, 28] in association with impending transition to MDD. However, besides low quality diagnostic measurement (via questionnaire-based self-reports, rather than clinical ratings) and the lack of control for confounding effects (e.g., due to pubertal changes or familial risk), these studies are drastically underpowered (e.g., average converter sample size of *n* = 22.55) [26]. Small sample sizes are hardly surprising as recruitment of converters is exceptionally challenging: considering the annual incidence of MDD of 4.3% [29], this requires the inclusion of large numbers of HC undergoing neuroimaging, alongside extensive long-term clinical follow-up assessments, to post-hoc identify an adequately sized sample of converters. Moreover, to the best of our knowledge, no available study has conducted a comparison of structural imaging data between converters and already affected patients with MDD in different remission states.

We faced this challenge by analyzing voxel-wise GMV of 45 initially healthy individuals with a subsequent transition to MDD (converters), healthy individuals who do not develop MDD within the next two years and a large heterogeneous sample of patients with MDD. Based on literature reporting that the dorsolateral prefrontal cortex (DLPFC), insula, rACC, as well as the hippocampus and amygdala, appear to be particularly affected in MDD, voxel-based morphometry was utilized in a region-of-interest (ROI) approach, complemented by exploratory whole-brain analyses. By contrasting converters to patients with MDD and HC, we aimed at disentangling GMV alterations in MDD as markers of vulnerability for an impending onset of an initial depressive episode and neural correlates of MDD that emerge later in the course of the disorder (analysis 1). To further investigate the impact of the acute depressive state (i.e., severe depressive symptoms during an acute depressive episode) on GMV, we subsequently only included patients in remission (analysis 2). Lastly, we compared converters with patients in remission and recurrence within the follow-up period to examine neural underpinnings of an *initial* depressive episode vs. a recurrent episode (analysis 3).

## Materials and methods

### Sample

The present study combined two large, independent longitudinal cohorts: the Marburg-Münster Affective Disorders Cohort Study (MACS) and the Münster Neuroimaging Cohort (MNC; Supplementary Methods 1). Both ongoing multimodal neuroimaging studies comprise converters, patients with affective, psychotic and anxious disorders and HC without transition to any mental disorder after follow-ups. Participants were aged between 18 and 65 years at baseline. For all participants, a magnetic resonance imaging (MRI) assessment and a consecutive clinical follow-up after approximately two years were considered. At the time of MRI assessment, both individuals in the converter sample and HC showed absence of any psychiatric diagnosis according to DSM-IV-TR but converters fulfilled the criteria for acute or lifetime MDD at the 2-year clinical follow-up. For details on the definition of groups and exclusion criteria see Supplementary Methods 2–3.

From the MACS, *n* = 1279 participants were analyzed, consisting of *n* = 30 converters, *n* = 590 patients with MDD and *n* = 659 HC. Patients diagnosed with MDD in the MACS cohort showed varying levels of symptom severity and underwent a range of treatments (inpatient, outpatient, or none). The MACS was conducted at two scanning-sites: University of Muenster and University of Marburg (see [30] for the general study description and [31] for MRI quality assurance protocol). From the MNC, *n* = 430 participants were included in this study, consisting of *n* = 15 converters, *n* = 158 patients with MDD and *n* = 257 HC. Patients were suffering from a moderate or severe depressive episode and underwent inpatient treatment at MRI assessment. The MACS was approved by the ethics committees of the Medical Faculties, University of Marburg (07/2014) and University of Muenster (2014-422-b-S). The MNC was approved by the ethics committee of the Medical Faculty of University of Muenster (2007-307-f-S).

The three groups did not differ regarding age or sex in the total sample (Table 1). Converters showed a significantly higher ratio of familial risk for MDD than HC but no differences in depressive symptoms. Compared to patients with MDD, converters experienced less depressive symptoms. Patients in the MNC reported a significantly higher number of lifetime in-patient treatments and higher levels of psychotropic medication intake compared to patients in the MACS (Table 2).

**Table 1:** Sample characteristics of the total sample, the remission sample and the recurrence sample by group.

	Total sample (*n* = 1709)							Remission sample (*n* = 1310)^a^				Recurrence sample (*n* = 1109)^b^			
Characteristic	CON (*n* = 45) *M (SD)*	HC (*n* = 916) *M (SD)*	MDD (*n* = 748) *M (SD)*	*p*^*1*^	CON vs. HC *p*^*2*^	CON vs. MDD *p*^*2*^	HC vs. MDD *p*^*2*^	MDD (*n* = 349) *M (SD)*	*p*^*1*^	CON vs. MDD *p*^*2*^	HC vs. MDD *p*^*2*^	MDD (*n* = 148) *M (SD)*	*p*^*1*^	CON vs. MDD *p*^*2*^	HC vs. MDD *p*^*2*^
Age	34.02 (11.46)	36.45 (13.04)	37.44 (13.29)	0.108	0.174	0.059	0.127	37.61 (13.23)	0.141	0.083	0.160	35.73 (13.44)	0.413	0.440	0.536
Gender (m/f) ^3^	18/27	372/544	273/475	0.227	0.935	0.636	0.087	112/237	**0.020**	0.288	0.**005**	44/104	**0.042**	0.196	**0.012**
Education level (y)^4^	14.80 (2.80)	14.20 (2.57)	13.56 (2.64)	**<0.001**	0.135	**0.003**	**<0.001**	13.67 (2.79)	**<0.001**	**0.010**	**<0.001**	13.20 (2.70)	**<0.001**	**<0.001**	**<0.001**
Cohort (MACS/ MNC) ^3^	30/15	659/257	590/158	0.002	0.443	0.054	**0.001**	344/5	**<0.001**	**<0.001**	**<0.001**	148/0	**<0.001**	**<0.001**	**<0.001**
HDRS Score	1.71 (2.50)	1.16 (1.73)	10.29 (7.41)	**<0.001**	0.151	**<0.001**	**<0.001**	4.99 (4.61)	**<0.001**	**<0.001**	**<0.001**	6.04 (4.72)	**<0.001**	**<0.001**	**<0.001**
Familial risk for MDD (no risk/risk) ^3^	31/14	786/124	553/189	**<0.001**	**0.001**	0.401	**<0.001**	234/114	**<0.001**	0.824	**<0.001**	99/49	**<0.001**	0.802	**<0.001**

*CON* converters, *HC* healthy controls, *MDD* major depressive disorder, *MACS* Marburg Münster Affective Disorders Cohort Study, *MNC* Münster Neuroimaging Cohort, *HDRS* Hamilton Depression Rating Scale.

^a^Characteristics of CON and HC see total sample. Statistic parameters of CON vs. HC comparison equals the total sample. Includes patients with remitted MDD only.

^b^Characteristics of CON and HC see total sample. Statistic parameters of CON vs. HC comparison equals the total sample. Includes patients with remitted MDD and recurrence within the 2-year follow-up interval only.

^1^MDD vs. CON vs. HC using one-way ANOVA except where noted.

^2^unpaired two-tailed *t* test except where noted.

^3^Χ²-test.

^4^Krustkal-Wallis-test.

Significant *p* values (*p* < 0.05) are highlighted in bold.

**Table 2:** Clinical characteristics of patients with MDD and converters for the total sample, the remission sample and the recurrence sample by study cohort.

	Patients with major depressive disorder											Converters		
	Total sample				Remission sample^a^				Recurrence sample^b^					
Characteristic	MACS (*n* = 590) *M* *(SD)*	MNC (*n* = 158) *M* *(SD)*	*p*^*1*^	MACS (*n* = 344) *M* *(SD)*		MNC (*n* = 5) *M* *(SD)*	*p*^*1*^	MACS (*n* = 148) *M* *(SD)*		MNC (*n* = 0) *M* *(SD)*	*p*^*1*^	MACS (*n* = 30) *M* *(SD)*	MNC (*n* = 15) *M* *(SD)*	*p*^*1*^
Time since onset (month)	131.92 (121.50)	128.09 (124.98)	0.739	137.31 (120.80)		NA	NA	143.19 (130.40)		NA	NA	NA	NA	NA
Number of depressive episodes lifetime	3.81 (6.58)	4.60 (5.79)	0.173	3.26 (5.29)		1.80 (4.03)	0.540	4.01 (6.74)		NA	NA	NA	NA	NA
Cumulative duration of depression (month)	43.61 (61.92)	34.97 (48.69)	0.070	38.16 (55.56)		24.00 (53.67)	0.572	46.86 (71.91)		NA	NA	NA	NA	NA
Number of in-patient treatments lifetime	1.48 (1.93)	2.13 (1.72)	**<0.001**	1.19 (1.67)		40 (0.89)	0.294	1.26 (1.50)		NA	NA	NA	NA	NA
Cumulative duration of in-patient treatment (weeks)	11.79 (18.10)	11.95 (16.03)	0.918	10.09 (17.65)		2.40 (5.37)	0.332	9.04 (10.71)		NA	NA	NA	NA	NA
Comorbidities (no/yes)^2^	336/253	78/80	0.085	209/135		4/1	0.381	80/68		NA	NA	NA	NA	NA
Medication Load Index	1.32 (1.49)	2.46 (1.70)	**<0.001**	95 (1.18)		40 (0.89)	0.295	1.05 (1.11)		NA	NA	NA	NA	NA
Time until onset (month)	NA	NA	NA	NA		NA	NA	NA		NA	NA	12.29 (7.29)	8.36 (7.22)	116

*MACS* Marburg Münster Affective Disorders Cohort Study, *MNC* Münster Neuroimaging Cohort.

^a^Includes partly or fully remitted patients only.

^b^Includes partly or fully remitted patients with recurrence within the 2-year follow-up interval only.

^1^MACS vs. MNC using unpaired two-tailed t-test except where noted.

^2^Χ²-test.

Significant *p* values (*p* <  0.05) are highlighted in bold.

### Materials and procedure

All participants underwent structural MRI. The Structural Clinical Interview for DSM-IV-TR (SCID-IV) [32] was conducted by trained personnel to verify presence or absence of psychiatric diagnosis at MRI assessment and clinical follow-up. At clinical follow-up, a life-chart method was used to accurately assess individual disease courses [33]. Current depression severity was assessed using the Hamilton Depression Rating Scale (HDRS [34];). To determine familial risk, participants were asked whether they had a first-degree relative who had been diagnosed with an affective disorder and received treatment for this. To account for psychotropic medication intake, a medication load index (MedIndex) was calculated as utilized in previous studies [17, 35, 36] (Supplementary Methods 4).

### Structural image acquisition and preprocessing

T1-weighted high-resolution anatomical images were acquired at 3 T MRI scanners through three-dimensional fast gradient echo sequences (Supplementary Methods 5). Within the MRI assessment period in the MACS cohort, the body-coil at the Marburg site was exchanged, which resulted in two dummy-coded variables considered by the applied harmonization algorithm. Further information on quality assurance protocol and scanner harmonization can be found elsewhere [31].

All T1-weighted images were preprocessed together using an identical processing and quality checking pipeline, employing the default parameter settings of the CAT12 toolbox (www.neuro.uni-jena.de/cat, v1720) implemented in the Statistical Parametric Mapping software (SPM12, version 7771, Institute of Neurology, London, UK). Briefly, processing steps included bias-correction, tissue segmentation, and spatially normalization to MNI-space using linear (12-parameter affine) and non-linear transformations, within a unified model including high-dimensional geodesic shooting normalization [37]. Gray matter images were smoothed with an 8 mm full width at half-maximum Gaussian kernel and underwent outlier detection using CAT12’s check homogeneity function and visual inspections. Data was harmonized using the batch correction tool ComBat [38] (Supplementary Methods 6).

### Statistical analyses

Analyses of clinical data and anomaly detection were calculated in R (https://www.R-project.org/). All second-level analyses of voxel-wise MRI data were performed with SPM12. We selected the rostral middle frontal gyrus, insula, rACC (subgenual and pregenual), amygdala, and hippocampus as ROIs and created separate bilateral masks according to the automated anatomical labeling atlas 3 (AAL3) [39]. The AAL3 is integrated into the Wake Forest University pickatlas [40] in SPM12. Following earlier studies [18, 19], the rostral middle frontal gyrus was chosen as representative of the DLPFC, since it encompasses Brodmann’s area 46 [41]. All statistical analyses were performed separately for each ROI. ROI analyses were conducted with a threshold-free cluster enhancement (TFCE) implemented in the TFCE-toolbox (http://dbm.neuro.uni-jena.de/tfce, Version222) with 5000 permutations per test and an absolute threshold masking of 0.1. The statistical threshold was set to a conservative familywise-error (FWE) correction of *p*_tfce-FWE_ < 0.05 on the voxel-level. For the main effect of group, Bonferroni correction was applied to account for multiple statistical tests across *n* = 5 ROIs (implying a threshold of *p*_tfce-FWE_ = .01). Exploratory whole-brain analyses were performed at a statistical threshold of *p* < 0.001, uncorrected, and a cluster threshold of *k* > 200 voxels.

**Analysis 1: vulnerability**. To investigate whether GMV changes represent a vulnerability, we performed full-factorial ANCOVAs on gray matter segments with group (converters vs. MDD vs. HC) as between-subjects factor and age, sex, and total intracranial volume (TIV) as covariates of no interest for each ROI separately and post-hoc two-sided *t* tests in case of significance. Any GMV alterations in converters compared to HC would indicate markers of the impending onset of MDD, representing either temporary vulnerability if emerging in converters only or rather stable pre-existing alterations if both converters and patients with MDD deviate from HC.

**Analysis 2: effects of acute depressive state**. To distinguish previously identified effects in MDD from effects of an acute depressive state (e.g., severe depressive symptoms during an acute depressive episode), significant between group differences (i.e., significant *t* tests) from analysis 1 were subsequently examined in a sample, which only comprised patients with MDD in partial or full remission (remission sample). Consequently, any remaining deviations in patients with remitted MDD from converters and HC would indicate rather persisting neural correlates of MDD.

**Analysis 3: first episode vs. recurrence**. To disentangle whether effects are present only before the first-time depressive episode or also prior a recurrent episode, significant between group differences (i.e., significant *t* tests) from analysis 2 were further examined in a sample that included patients with currently remitted MDD, who furthermore experienced recurrence within the follow-up interval (recurrence sample). A recurrent episode was assessed the identical way as the converters’ first episode within the follow-up interval.

**Additional Analyses**. To address potential impact of large size differences of the subgroups on significant results, we additionally repeated the procedure described in analysis 3 in a smaller sample matched for age, sex, and scanner variables in a last step (Supplementary Methods 7 and Tables 1–2). Further checks on validation of distributional assumptions and sanity can be found in the Supplementary Methods 8. Lastly, we conducted anomaly detection using an Isolation Forest approach to provide a more nuanced analysis of the data, capturing individual variability while transcending group-level differences (Supplementary Methods 9).

## Results

### Analysis 1: vulnerability

**Fig. 1:**
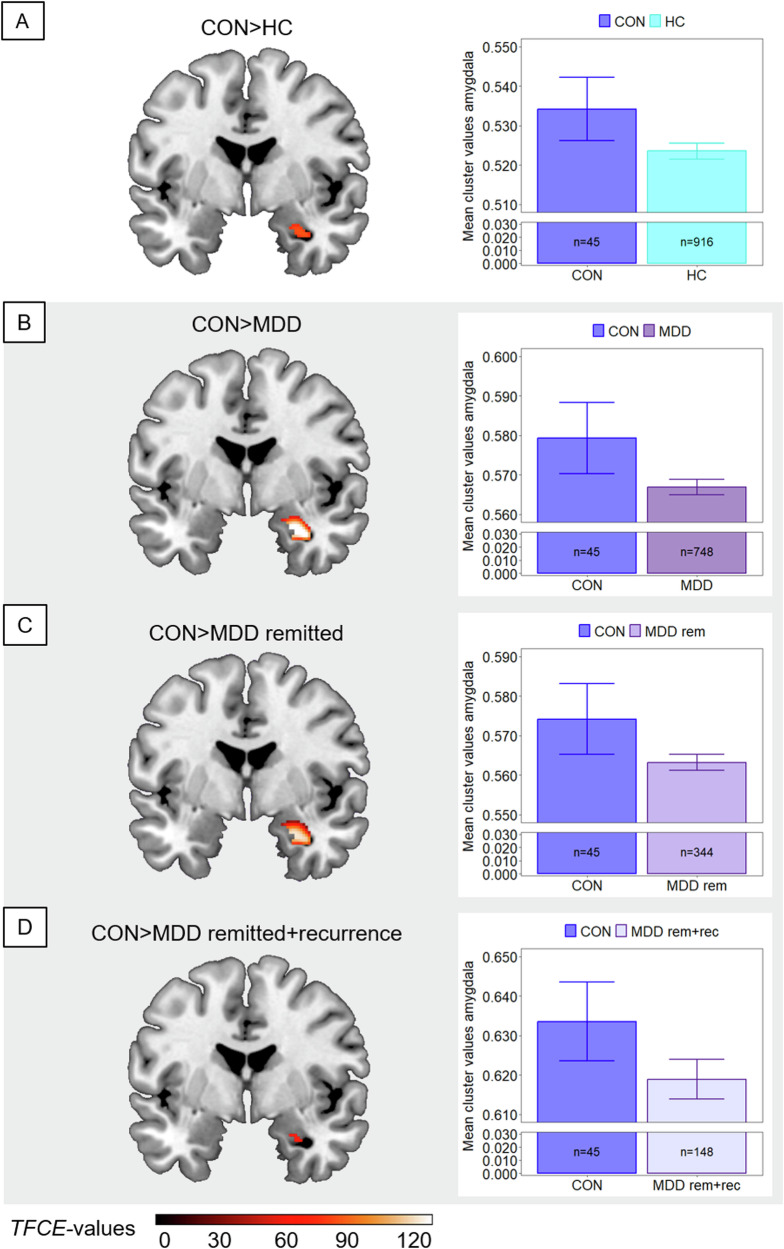
Larger gray matter volumes in the amygdala in converters compared to HC and patients with MDD in different remission states. Comparisons between converters (CON) and subgroups are shown. Clusters are thresholded at *p*_tfce-FWE_ < 0.05 and bar plots represent respective extracted Eigenvariates from the depicted cluster. Error bars indicate 1 SEM. *T*-contrasts are shown for the comparison of converters with (**A**) healthy controls (HC), (**B**) patients with major depressive disorder (MDD) in heterogeneous remission states, (**C**) patients with currently remitted MDD (MDD rem), and (**D**) patients with currently remitted MDD with recurrence within a 2-year clinical follow-up period (MDD rem+rec). Shaded subplots indicate comparisons of converters with MDD subgroups.

ROI analyses in the DLPFC, insula and amygdala revealed significant main effects of group (all *p*_tfce-FWE_ < 0.048, partial η²>0.007), however findings in the DLPFC and insula did not survive Bonferroni correction. No main effect of group emerged in the rACC (*p*_tfce-FWE_ = 0.184) and hippocampus (*p*_tfce-FWE_ = 0.142). While the significant effects were mainly driven by lower GMV in patients with MDD compared to HC (all *p*_tfce-FWE_ < 0.010, *d* > 0.186) in the DLPFC and insula (Supplementary Fig. 1), we found significantly higher amygdala volumes in converters compared to both HC (*t*(1703) = 2.93, *p*_tfce-FWE_ = 0.037, Cohen’s *d* = 0.447; Fig. 1A) and patients with MDD (*t*(1703) = 3.31, *p*_tfce-FWE_ = 0.005, Cohen’s *d* = 0.508, Fig. 1B). In the DLPFC and insula, no differences emerged between patients with MDD compared to converters or in converters compared to HC in post-hoc *t* tests (Supplementary Table 3).

### Analysis 2: effects of acute depressive state

In ROI analyses in the remission sample, the *t* tests did not reveal lower GMV in the DLPFC (*p*_tfce-FEW_ = 0.114) and insula (*p*_tfce-FWE_ = 0.175) in patients with remitted MDD compared to HC. In the amygdala, however, converters showed more GMV compared to patients with remitted MDD (*t*(1299) = 3.17, *p*_tfce-FWE_ = 0.002, Cohen’s *d* = 0.502, Fig. 1C) and compared to HC (*t*(1299) = 2.86, *p*_tfce-FWE_ = 0.014, Cohen’s *d* = 0.437). For details, see Supplementary Table 4.

### Analysis 3: first episode vs. recurrence

Since in analysis 2, we found significant effects only in the amygdala, we repeated the ROI analysis in the amygdala in the recurrence sample. Again, the *t* tests revealed larger volumes in the amygdala in converters compared to patients with remitted MDD and recurrence within the follow-up interval (*t*(1103) = 2.67, *p*_tfce-FWE_ = 0.046, Cohen’s *d* = 0.455; Fig. 1D) as well as compared to HC (*t*(1103) = 2.83, *p*_tfce-FWE_ = 0.024, Cohen’s *d* = 0.432). There were no significantly lower amygdala volumes in patients compared to HC (*p*_tfce-FWE_ = 0.629; Supplementary Table 5).

### Additional analyses

Findings from exploratory whole-brain analyses in the three samples concordant with reported effects from the ROI analyses in the DLPFC and insula in clusters comprising e.g., temporal and frontal regions, thalamus and insula (Supplementary Table 6).

For detailed results on sanity checks see Supplementary Tables 7–10 and Figs. 2–4. Briefly, the amygdala effect remained significant after Bonferroni correction for multiple testing, independent of added covariates, and in the smaller sample matched for age, sex, and scanner variables. After controlling for familial risk for MDD and education level, group differences described in analysis 1 remained significant. When adding medication as covariate, remaining significant effects were comparable to results described in analysis 2. Our Isolation Forest approach applied on GMV of the amygdala identified converters with a higher percentage (CON: 24.4%, MDD: 8.7%, HC: 8.0%, χ²(2) = 14.63, *p* < 0.001) as abnormal compared to HC and patients (Supplementary Fig. 5).

## Discussion

This study examined GMV differences among healthy individuals transitioning to MDD (converters), patients with MDD in various remission states, and HC with no depressive episodes during follow-up. Aim of the study was to disentangle pre-existing markers of neurobiological vulnerability for the future onset of a depressive episode and correlates of MDD emerging in already affected individuals, especially focusing on the impact of the acute depressive state. Two main findings emerged: firstly, converters showed more amygdala GMV compared to patients with MDD and HC. This effect remained significant when contrasting converters with remitted patients with recurrence, suggesting the amygdala enlargement is a temporary marker for the future onset of MDD. Secondly, less volumes in the DLPFC and insula in patients most likely do not represent depression vulnerability and are at least partially driven by the acute depressive state.

We found more GMV in the amygdala in converters relative to both patients and HC. The converter sample comprises currently healthy individuals who do not yet show elevated levels of depressive symptoms compared to HC but are known to experience their first-time depressive episode within the next two years. Thus, the amygdala enlargement neither reflects a state effect associated to acute depressive symptoms nor a persistent neural correlate of depression. It might rather represent a temporary vulnerable state that marks the onset of an impending first depressive episode. While findings on amygdala GMV in depression onset [26] and recurrence [23, 42] are inconsistent, our results suggest it tracks the dynamic course of the disorder, which might reflect its key role in emotion regulation and stress response [9, 43]. To date, the precise trajectory of these alterations and their associations with further risk factors (e.g., environmental factors) remains unclear. Observations of more amygdala GMV in converters relative to HC align with Nickson et al. (2016). Although their study lacked a direct comparison with a clinical MDD sample, they reported a decline in amygdala volume over time in converters, a pattern matching our findings of reduced amygdala volumes within patients. Research in patients with acute first episode depression frequently reported enlarged amygdala volumes [21, 22], including comparisons with recurrent MDD [44]. Taken together with our findings from contrasting converters and remitted patients with recurrence within follow-up, the amygdala enlargement may be a neural marker of the impending *onset* of MDD, rather than a recurrent episode. Potentially, this could be associated with enhanced metabolism in the amygdala and persistent hyperactivity to negative stimuli in MDD [45, 46]. Another possible mechanism behind the amygdala enlargement might be stress-induced inflammatory processes such as microglia and astrocyte activation [47, 48]. Once activated, these cells proliferate and undergo morphological changes such as cell body enlargement [49], which might influence GMV change [50]. As the disease progresses, stress-related neurotoxic processes [51–54] during recurrent depressive episodes may lead to a subsequent decline in GMV. Alterations related to disease stage may explain the heterogeneous findings on amygdala volume. Additionally, the complex structure and lateralization of the amygdaloid region may also contribute. In our study, we observed higher volumes in the right amygdala in converters. This finding aligns with two recent reviews [10, 55] highlighting rightward structural asymmetry in MDD and supports the theory that the right hemisphere plays a dominant role in processing negative emotions [56]. Notably, however, the link between functional abnormalities and structural changes remains inconclusive, with several studies reporting no consistent association [57].

Our findings on lower GMV in the DLPFC and insula ROIs in patients with MDD, but also in frontal and temporal regions that emerged in whole-brain analyses, do not seem to indicate vulnerability for MDD, since we did not observe such alterations in the comparison of converters and HC. Furthermore, after correcting for multiple statistical tests or restricting the analysis to patients with currently remitted MDD, the observed effects were no longer significant. Following this, lower volumes in the DLPFC and insula may reflect the acute depressive state and partially normalize with remission rather than being a persistent neural correlate of MDD. In our recent work on replicability and generalizability of gray matter alterations in MDD we found a rather similar pattern of GMV reductions across stratified subsamples of patients with MDD (e.g., acute/remitted, medicated/non-medicated) implying a general MDD diagnosis effect [7]. Since our remission sample was restricted to a lower sample size (*n* = 349), our results could be driven by insufficient statistical power considering small effect sizes in neuroimaging modalities [58]. Nevertheless, replicability was highest when contrasting HC with acutely depressed patients [7], which could be related to effects of the acute depressive state to some extent. Previous cross-sectional [15, 59, 60] and longitudinal research [20, 61] on volumes of DLPFC and insula suggested neural correlates of MDD might actually represent a persistent, even scar-like effect, as stronger GMV decline was associated with more severe disease courses (e.g., duration of disease or number of episodes). However, evidence on such scar-like effects is limited due to cross-sectional study designs and in general the inadequately powered sample sizes of patient (sub)groups coming with a risk for inflated effects [62]. In line with our results, longitudinal studies found GMV reductions in patients to disappear with statistical control for influence of current depression severity and that GMV in times of remission either remained unchanged while it decreased in non-remitters [17, 19] or even increased [18]. Additional preliminary evidence on the impact of acute depressive state on the brain comes from recent studies in other neuroimaging modalities such as task-based fMRI [63] and structural connectome analysis [64]. Less severe depressive symptoms, e.g., in times of remission, might be associated with less subjective stress exposure, related to the inhibition of stress-mediated neurotoxic processes in depression [51–54] in structures of the frontolimbic circuitry. Following this, presumably increased stress levels in converters before they experience their first-time depressive episode might also explain the absence of volume differences in the DLPFC or insula between converters and patients with MDD as well as between converters and HC. Potentially, an incipient GMV decline in these reciprocally connected structures [65] may be present albeit too small to achieve statistical significance in the converter sample.

Regarding GMV of the hippocampus and rACC, we did not find any significant effects between the groups, which stands in contrast to earlier research [1–3]. Nevertheless, our null-findings for the hippocampus are consistent with two recent studies both conducted in samples comprising over 4000 individuals [7, 66]. For the rACC, it is consistently discussed as a biomarker to predict treatment outcomes and a good clinical course (for a review see [67]). It therefore seems consistent with our findings that changes in ACC are not per se a vulnerability or state marker of depression. Rather, it may play a role in facilitating treatment response and subsequent remission. Future studies should investigate this in more detail.

The major strength of this study is the unprecedented large sample of individuals transitioning to MDD, which required the recruitment of a large number of HC undergoing neuroimaging with additional clinical follow-up assessments. Data of this valuable population is crucial to make assumptions about neuronal vulnerability for the onset of MDD. Contrasting converters and HC with patients in different states of remission also expanded our understanding of the impact of the acute depressive state on GMV. Further, this study compares the morphometric characteristics of an impending *first* depressive episode with those of an impending *recurrent* episode. Moreover, depressive episodes were verified with the SCID-IV [32] by trained raters, which significantly increases the data quality compared to diagnoses based on self-report.

A few limitations need to be addressed. Firstly, the cross-sectional design limits conclusions about higher amygdala GMV as a transient vulnerability marker for MDD onset. As our converter sample was too small to perform predictive machine learning-based approaches [68], we included anomaly detection using Isolation Forest to capture individual variability beyond group-level analyses. This approach revealed a three times higher abnormality percentage in converters compared to HC and patients. Secondly, the subgroups strongly differed in size and the converter sample size is insufficient to detect small effects common in neuroimaging modalities [58]. To adjust for this, we matched the groups by sex, age, and scanner, and employed a dismantling approach with additional sanity checks to gradually eliminate confounding factors. To our knowledge, our converter sample (*n* = 45) is among the largest so far [26, 69]. Thirdly, the time until onset of MDD in converters varied between one and 24 months, limiting conclusions on the exact timing of morphometric changes, though GMV changes in the amygdala, insula, and DLPFC were not linked with the months until onset. Fourthly, due to the 2-year follow-up period, we cannot definitively rule out the possibility that individuals in the HC subgroup may develop MDD in the future, and they should therefore technically be considered part of the converter subgroup. The potential influence of these converters should still be taken into account when interpreting the results. Lastly, we combined two clinical cohorts using different scanners to increase sample size. Patients from the MNC cohort were predominantly acutely depressed patients, thus the remission and recurrence sample mainly included patients from the MACS cohort. To address potential scanner effects in these samples, data were harmonized using ComBat [38] prior excluding acute cases.

In conclusion, examining one of the largest available converter samples in neuroimaging research offered preliminary evidence on neural markers for the impending onset of an initial depressive episode and other correlates of MDD that emerge later in the course of the disorder and are probably driven by the acute depressive state. Our findings extend neurobiological models of the etiology of MDD [4, 5, 43, 70] and suggest a temporary vulnerability, which in combination with other common risk factors for MDD might facilitate prediction of the onset of the disorder. In turn, this could improve both early detection and prevention programs for depression. Finally, encouragement is given for longitudinal studies focusing on such highly relevant converter samples prior to, during, and following the initial depressive episode to further elucidate GMV trajectories in the early stages of depression.

### Data access and responsibility

FOR2107/MACS cohort data: All PIs take responsibility for the integrity of the respective study data and their components. All authors and coauthors had full access to all study data. MNC data: AK and UD take responsibility for the integrity of the data and the accuracy of data analysis.

### Previously published work

Both datasets are part of a larger consortium and have already been published numerous times (please see https://for2107.de/publikationen/ for the MACS and https://www.medizin.uni-muenster.de/translap/studien/nae.html for the MNC). This manuscript presents the first analysis of gray matter volumes of initially healthy individuals with a subsequent transition to major depressive disorder (converters). We compared these to both, patients with major depressive disorder and sustained healthy controls. To increase the sample size, especially of the converter subsample, we combined datasets of the MACS and the MNC. For the first time, we included only individuals with available gray matter data from the MRI assessment who also completed the 2-year clinical follow-up assessment from both cohorts.

## Supplementary information

Supplementary Material
