# Supplementary material for: Brain structural correlates of an impending initial major depressive episode
Source: Neuropsychopharmacology. 2025 Mar 12;50(7):1176–85. doi: 10.1038/s41386-025-02075-6 (PMC12089404; doi:10.1038/s41386-025-02075-6)
Supplement: Supplementary file 1 — Supplementary Material [file 41386_2025_2075_MOESM1_ESM.docx]

**Supplementary Material**

## Supplementary Methods

### Supplementary Methods 1: Description of the cohorts

We included two cohorts in this study. The Marburg-Münster Affective Disorders Cohort Study (MACS) is a bicentric epidemiological study from Marburg and Münster funded by the DFG FOR2107 [1]. It enrolled more than n=2500 healthy controls as well as patients with lifetime diagnoses of affective, psychotic and anxious disorders and collected detailed phenotypical information, biomaterial and multimodal neuroimaging data. The participants from the MACS are follow-uped every two to three years. The study started in 2013 and the third follow-up is still ongoing. Participants were recruited through local psychiatric hospitals or newspaper advertisements. MRI data collection for this study took place between September 2014 and June 2019. The MACS was approved by the Ethics Committees of the Medical Faculties, University of Marburg (07/2014) and University of Muenster (2014-422-b-S).

The Münster Neuroimaging Cohort (MNC) focuses on the neurobiology of affective disorders and its trajectories through longitudinal assessments. Detailed phenotypical information, biomaterial and multimodal neuroimaging data is collected at baseline and each follow-up. Participants are re-invited approximately every two years and follow-ups are still ongoing. The study started in 2009 resulting in up to seven follow-ups per person. Patients with major depressive disorder (MDD) were recruited from the inpatient service of two local psychiatric hospitals. Recruitment of healthy controls (HC) was carried out through public notices and newspaper announcements. MRI data for this study was acquired between October 2009 and December 2020. The MNC was approved by the ethics committee of the Medical Faculty of University of Muenster (2007-307-f-S). All participants provided written informed consent and received financial compensation.

### Supplementary Methods 2: Definition of groups

In order to maximize the sample size, the baseline data, i.e. the data at first inclusion in the MACS or MNC, was selected for the HC and MDD subgroups. The converter group consists of participants who were initially diagnosed as HC but experienced their first depressive episode at some point before their repeated follow-up assessments. Consequently, the occurrence of a first depressive episode during the study interval was used to retrospectively categorize individuals initially recruited as HC into either the converter group or the sustained HC group. To examine proximal neurobiological alterations (in addition to stable predispositions) preceding this critical change of diagnosis, we examined the assessment immediately preceding the first depressive episode of converters. For a participant who joined the study in 2010 (baseline) as a healthy control who reported their first depressive episode 8-years later in 2018 (4th follow-up), we included MRI data from their 3rd visit 6 years after the initial study inclusion. In addition, the use of baseline data for converters would introduce high variability in the time between baseline and episode onset, which would increase the likelihood of confounding effects from unrelated factors such as life events or aging.

### Supplementary Methods 3: Exclusion criteria

Any lifetime diagnoses of schizophrenia, schizoaffective disorder, bipolar disorder, or substance dependence according to DSM-IV [2] were excluded. Further exclusion criteria were neurological abnormalities, a history of seizures, head trauma or unconsciousness, severe physical impairments (e.g., cancer or epilepsy), hypothyroidism without proper medication, claustrophobia, color blindness, and general magnetic resonance imaging (MRI) contradictions (such as ferromagnetic implants or pregnancy).

### Supplementary Methods 4: Computation of medication load index

Type and dose of psychopharmacological treatment were assessed in structured clinical interviews. First, to measure the total medication load, a strategy described earlier was used [3]. Each psychotropic medication was coded as absent=0, low=1 (equal or lower average dose), or high=2 (greater than average dose), relative to the midpoint of the daily dose range recommended by Physician’s-Desk-Reference [4]. A composite measure of total medication load was calculated for each individual, reflecting dose and variety of different medications taken, by summing all individual medication. Next, the mean of the total medication scores was calculated at both time-points. Finally, this total medication load mean was multiplied with the percentage of days receiving medication during follow-up, to integrate days without the influence of psychopharmacological treatment.

### Supplementary Methods 5: Detailed description of MRI parameters

In the MACS, T1-weighted high-resolution anatomical data were collected at 3T MRI scanners using three-dimensional (3D) fast gradient echo sequences (MPRAGE) recorded by 3T MRI scanners (Marburg: Tim Trio, Siemens, Erlangen, Germany; Münster: Prisma, Siemens, Erlangen, Germany). Sequence parameters were: 176 sagittal slices, slice gap 0.5mm, TR=1900ms, TE=2.26ms, inversion time=900ms, FA=9°, voxel size=1x1x1mm³ (Marburg) and 192 sagittal slices, slice gap 0.5mm, TR=2130ms, TE=2.28ms, inversion time=900ms, FA=8°, voxel size=1x1x1mm³ (Münster). During data acquisition, the body- coil in Marburg was exchanged resulting in three different scanner settings for all analyses (Marburg body- coil pre, Marburg body- coil post and Münster) [5].

In the MNC, 1-weighted high-resolution anatomical images of the head were acquired (Gyroscan Intera 3T, Philips Medical Systems, the Netherlands) using a three-dimensional fast gradient echo sequence (turbo field echo), repetition time=7.4 ms, echo time=3.4 ms, flip angle=9°, two signal averages, inversion prepulse every 814.5 ms, acquired over a field of view of 256 mm (feet-head) x 204 mm (anterior-posterior) x 160 mm (right-left), frequency encoding in feet to head direction, phase encoding in anterior-posterior and right-left direction, reconstructed to voxels of 0.5 mm × 0.5 mm × 0.5 mm.

### Supplementary Methods 6: ComBat Harmonization

To adjust for scanner and site effects, data was harmonized using the MatLab version of ComBat (<https://github.com/Jfortin1/ComBatHarmonization/tree/master/Matlab>). ComBat, a popular batch correction tool, has been applied in previous neuroimaging studies using different brain structural modalities [6–8]. Using an empirical Bayesian framework, ComBat successfully removes variability attributed to scanner and site differences [9,10].

### Supplementary Methods 7: Matched sample

To verify whether potential brain structural alterations are present only before the first ever depressive episode or also prior a recurrent episode, we additionally created a smaller sample (*n*=135) matched for age, sex, and scanner variables within each cohort separately (Supplementary Table 1-2). Regarding scanner variables, in the MACS cohort we matched for site (Marburg vs. Münster) and body coil change (pre vs. post body coil change). In the MNC, we matched for the year of MRI assessment, since this study is ongoing since 2009 and includes up to seven follow-up assessments per person. Thus this sample included baseline or follow-up data of HC and converters, depending on the time of onset of MDD. Since patients in the MNC were all suffering from an acute moderate or severe depressive episode when initially recruited, the matched sample only included follow-up data of patients who were in remission at time of assessment. Again, data was harmonized using ComBat (<https://github.com/Jfortin1/ComBatHarmonization/tree/master/Matlab>).

### Supplementary Methods 8: Checks on distributional assumptions and sanity

We assessed the residuals of gray matter data for each ROI to ensure the validity of the distributional assumptions. To evaluate normality, we conducted Kolmogorov-Smirnov tests (Supplementary Table 10), complemented by visual inspection of Q-Q plots (Supplementary Figure 2).

In the converter group, we found a significantly higher ratio of familial risk for MDD compared to HC. Thus, besides controlling for psychopharmacological treatment, we further repeated the ANCOVAs from analysis 1 separately with familial risk for MDD as additional covariate to exclude confounding effects (Supplementary Table 9). Additionally, given the significantly higher education levels observed in both converters and healthy controls compared to patients, we included years of education as a covariate in our analyses (Supplementary Table 9). The statistical threshold was set to a conservative familywise-error (FWE) correction of p < 0.05 on the voxel-level, without cluster extent threshold. Moreover, we analyzed whether higher gray matter volumes (GMV) in the amygdala in converters were associated with the time until the onset of the initial depressive episode (Supplementary Figure 3). To check for influential data points, we used boxplots. We plotted the residuals from the linear model, adjusting the GMV from the amygdala peak voxel derived from the significant cluster identified in analysis 1 (x = 30, y = -3, z = -24). The boxplots did not reveal many outliers in the data (Supplementary Figure 4).

### Supplementary Methods 9: Anomaly detection with Isolation Forest

We performed anomaly detection using an Isolation Forest approach to provide a more nuanced analysis of the data, capturing individual variability while transcending group-level differences. The Isolation Forest algorithm is a tree-based unsupervised method designed to detect anomalies by isolating data points in a feature space, implemented in the R package “isotree” [11]. The analysis was performed across all participants on gray matter values extracted from the significant amygdala cluster of analysis 1 at peak voxel level (x = 30, y = -3, z = -24). The model was trained using 500 trees, with each tree constructed using a random subsample of the data. The sample size per tree was set to *n*=854 (approximately 50% of the total dataset size of *n*=1709). Anomaly scores were computed for each individual, representing the likelihood of being an outlier based on the data's distribution. A threshold was applied to classify anomalies, defined as the top 10% of anomaly scores. Individuals exceeding this threshold were labeled as anomalies ("Yes"), while the remaining were classified as non-anomalous ("No"). Lastly, we calculated the proportion of anomalies as a percentage for each group and performed a Chi-squared test to assess whether the distribution of anomalies differed significantly across the groups.

### Supplementary Table 1

*Sample characteristics of the smaller sample matched for sex, age, and scanner variables divided by group*

| Characteristic | CON  (*n*=45)  *M (SD)* | HC  (*n*=45)  *M (SD)* | MDD  (*n*=45)  *M (SD)* | *p^1^* | CON  vs.  HC  *p^2^* | CON  vs.  MDD  *p^2^* | HC  vs.  MDD  *p^2^* |
| --- | --- | --- | --- | --- | --- | --- | --- |
| Age | 34.02  (11.46) | 37.31  (13.93) | 35.60  (12.45) | .470 | .225 | .531 | .543 |
| Gender (m/f)^3^ | 18/27 | 21/24 | 20/25 | .810 | .523 | .670 | 1.000 |
| Cohort (MACS/MNC)^3^ | 30/15 | 30/15 | 30/15 | 1.000 | 1.000 | 1.000 | 1.000 |
| Education  level (y)^4^ | 14.80  (2.80) | 13.84  (2.37) | 13.33  (3.02) | **.033** | .091 | **.016** | .206 |
| HDRS Score | 1.71  (2.50) | 1.09  (2.25) | 6.69  (5.25) | **<.001** | .218 | **<.001** | **<.001** |
| Familial risk for MDD  (no risk/risk)^3^ | 31/14 | 45/0 | 29/16 | **<.001** | **<.001** | .655 | **<.001** |

*Note.* CON=converters, HC=healthy controls, MDD=major depressive disorder, MACS=Marburg Münster Affective Disorders Cohort Study, MNC=Münster Neuroimaging Cohort, HDRS=Hamilton Depression Rating Scale. Significant p-values (*p* < .05) are highlighted in bold. The matched sample includes remitted patients only who experienced a relapse within a 2-year follow-up interval.

^1^ MDD vs. CON vs. HC using one-way ANOVA except where noted.

^2^ unpaired two-tailed t-test except where noted.

^3^ Χ²-test.

^4^ Krustkal-Wallis-test.

### Supplementary Table 2

*Clinical characteristics of patients with MDD and converters for the smaller sample matched for sex, age, and scanner variables divided by study cohort*

|  | Patients with MDD | | | | | Converters | | |
| --- | --- | --- | --- | --- | --- | --- | --- | --- |
|  |  |  | |  |  |  |  |  |
| Characteristic | MACS  (*n*=30)  *M*  *(SD)* | | MNC  (*n*=15)  *M*  *(SD)* | | *p^1^* | MACS  (*n*=30)  *M*  *(SD)* | MNC  (*n*=15)  *M*  *(SD)* | *p^1^* |
| Time since onset (month) | 162.80 (120.69) | | 99.66 (58.04) | | **.022** | NA | NA | NA |
| Number of depressive episodes lifetime | 4.08 (3.97) | | 4.33 (2.69) | | .828 | NA | NA | NA |
| Cumulative duration of depression (month) | 74.05 (100.20) | | 25.17 (21.45) | | .**046** | NA | NA | NA |
| Number of in-patient treatments lifetime | 1.43 (1.59) | | 2.00 (1.25) | | .235 | NA | NA | NA |
| Cumulative duration of in-patient treatment (weeks) | 10.60 (11.46) | | 14.60 (23.67) | | .544 | NA | NA | NA |
| Comorbidities (no/yes)^2^ | 14/16 | | 5/10 | | .393 | NA | NA | NA |
| Medication Load Index | 1.20 (1.19) | | 1.20 (1.37) | | 1.000 | NA | NA | NA |
| Time until onset (month) | NA | | NA | | NA | 12.29  (7.29) | 8.36  (7.22) | .116 |

*Note.* MACS=Marburg Münster Affective Disorders Cohort Study, MNC=Münster Neuroimaging Cohort MDD=major depressive disorder. Significant p-values (*p* < .05) are highlighted in bold. The matched sample includes remitted patients only who experienced a relapse within a 2-year follow-up interval.

^1^ MACS vs. MNC using unpaired two-tailed t-test except where noted.

^2^ Χ²-test

## Supplementary Results

### Supplementary Table 3

*Results from ANCOVAs and t-tests (analyses 1) in the total sample (n=1709) for the dorsolateral prefrontal cortex, insula and amygdala*

|  |  |  | Peak voxel coordinates | | |  |  |  |
| --- | --- | --- | --- | --- | --- | --- | --- | --- |
| Contrast | Side | Cluster size | x | y | z | *F-/t-*value | *p_tfce-FWE_*-value | Effect size  partial η²/*d* |
|  | Dorsolateral prefrontal cortex | | | | | | | |
| Main effect group | R | 92 | 34 | 44 | 30 | 9.37 | .**032** | .011 |
| HC>MDD | R | 825 | 36 | 44 | 32 | 3.81 | **.006** | .188 |
|  | R | 37 | 32 | 48 | 16 | 3.41 | **.043** | .168 |
| HC<MDD | - | - | - | - | - | - | .643 | - |
| CON>MDD | - | - | - | - | - | - | .302 | - |
| CON<MDD | - | - | - | - | - | - | .348 | - |
| HC>CON | - | - | - | - | - | - | .157 | - |
| HC<CON | - | - | - | - | - | - | >.999 | - |
|  | Insula | | | | | | | |
| Main effect group | R | 14 | 45 | 0 | 2 | 7.40 | .**048** | .009 |
| HC>MDD | R | 703 | 44 | 0 | 2 | 3.78 | **.010** | .186 |
| HC<MDD | - | - | - | - | - | - | .677 | - |
| CON>MDD | - | - | - | - | - | - | .067 | - |
| CON<MDD | - | - | - | - | - | - | .367 | - |
| HC>CON | - | - | - | - | - | - | .170 | - |
| HC<CON | - | - | - | - | - | - | .360 | - |
|  | Amygdala | | | | | | | |
| Main effect group | R | 156 | 30 | -3 | -24 | 5.71 | .**007*** | .007 |
| HC>MDD | L | 3 | -21 | 0 | -16 | 2.73 | .**049** | .135 |
| HC<MDD | - | - | - | - | - | - | >.999 | - |
| CON>MDD | R | 283 | 30 | -3 | -24 | 3.31 | **.005** | 0.508 |
| CON<MDD | - | - | - | - | - | - | >.999 | - |
| HC>CON | - | - | - | - | - | - | .195 | - |
| HC<CON | R | 64 | 30 | -3 | -24 | 2.93 | **.037** | 0.447 |

*Note.* HC=healthy controls, CON=converters, MDD=major depressive disorder. Significant *p*-values of the significance level *p* < .05 are highlighted in bold.

*Significant after Bonferroni correction for multiple statistical tests (*p*=.01)

### Supplementary Table 4

*Results from ANCOVAs and t-tests (analyses 2) in the remission sample (n=1310) for the dorsolateral prefrontal cortex, insula and amygdala*

|  |  |  | Peak voxel coordinates | | |  |  |  |
| --- | --- | --- | --- | --- | --- | --- | --- | --- |
| Contrast | Side | Cluster size | x | y | z | *F-/t-*value | *p_tfce-FWE_*-value | Effect size partial  η²/*d* |
|  | Dorsolateral prefrontal cortex | | | | | | | |
| Main effect group | - | - | - | - | - | - | .300 | - |
| HC>MDD | - | - | - | - | - | - | .114 | - |
| HC<MDD | - | - | - | - | - | - | .548 | - |
| CON>MDD | - | - | - | - | - | - | .253 | - |
| CON<MDD | - | - | - | - | - | - | .361 | - |
| HC>CON | - | - | - | - | - | - | .164 | - |
| HC<CON | - | - | - | - | - | - | .905 | - |
|  | Insula | | | | | | | |
| Main effect group | - | - | - | - | - | - | .288 | - |
| HC>MDD | - | - | - | - | - | - | .175 | - |
| HC<MDD | - | - | - | - | - | - | .630 | - |
| CON>MDD | - | - | - | - | - | - | .061 | - |
| CON<MDD | - | - | - | - | - | - | .588 | - |
| HC>CON | - | - | - | - | - | - | .159 | - |
| HC<CON | - | - | - | - | - | - | .267 | - |
|  | Amygdala | | | | | | | |
| Main effect group | - | - | - | - | - | - | .083 | - |
| HC>MDD | - | - | - | - | - | - | .146 | - |
| HC<MDD | - | - | - | - | - | - | >.999 | - |
| CON>MDD | R | 316 | 28 | -3 | -24 | 3.17 | **.002** | **.012** |
| CON<MDD | - | - | - | - | - | - | .747 | - |
| HC>CON | - | - | - | - | - | - | .194 | - |
| HC<CON | R | 189 | 30 | -3 | -24 | 2.86 | **.014** | **.042** |

*Note.* HC=healthy controls, CON=converters, MDD=major depressive disorder. Significant *p*-values of the significance level *p* < .05 are highlighted in bold.

### Supplementary Table 5

*Results from ANCOVA and t-tests (analyses 3) in the recurrence sample (n=1109) for the amygdala*

|  |  |  | Peak voxel coordinates | | |  |  |  |
| --- | --- | --- | --- | --- | --- | --- | --- | --- |
| Contrast | Side | Cluster size | x | y | z | *F-/t-*value | *p_tfce-FWE_*-value | Effect size partial  η²/*d* |
| Main effect group | - | - | - | - | - | - | .151 | - |
| HC>MDD | - | - | - | - | - | - | .684 | - |
| HC<MDD | - | - | - | - | - | - | >.999 | - |
| CON>MDD | R | 23 | 28 | -3 | -24 | 2.67 | .046 | .455 |
| CON<MDD | - | - | - | - | - | - | .589 | - |
| HC>CON | - | - | - | - | - | - | .240 | - |
| HC<CON | R | 146 | 28 | -3 | -24 | 2.83 | .024 | .432 |

*Note.* HC=healthy controls, CON=converters, MDD=major depressive disorder. Significant *p*-values of the significance level *p* < .05 are highlighted in bold.

### Supplementary Table 6

*Results from exploratory whole-brain analyses in the three samples conducted at p < .001, uncorrected, with a cluster threshold of k=200*

|  |  |  | | Peak voxel coordinates | | | | |  | |  |
| --- | --- | --- | --- | --- | --- | --- | --- | --- | --- | --- | --- |
| Anatomical region | Side | | Cluster size^1^ | | x | y | z | *t-/F-*value | | *p_unc_*-value | |
|  | Total sample (*n*=1709) | | | | | | | | | | |
| *Main effect group* |  | |  | |  |  |  |  | |  | |
| Middle temporal gyrus, superior temporal gyrus | R | | 741 | | 56 | -20 | -14 | 12.5 | | **<.001** | |
| Angular gyrus, middle occipital gyrus | L | | 486 | | -46 | -70 | 33 | 10.69 | | **<.001** | |
|  |  | | 271 | | -56 | -68 | 15 | 9.21 | | **<.001** | |
| *HC>MDD* |  | |  | |  |  |  |  | |  | |
| Inferior temporal gyrus | L | | 474 | | -52 | -24 | -27 | 4.42 | | **<.001** | |
| Middle temporal gyrus, superior temporal gyrus, middle temporal pole | R | | 1142 | | 56 | -21 | -12 | 4.41 | | **<.001** | |
| Angular gyrus, middle occipital gyrus, inferior parietal gyrus | L | | 710 | | -46 | -69 | 33 | 4.19 | | **<.001** | |
| Middle temporal gyrus, superior temporal gyrus | L | | 615 | | -51 | -24 | -15 | 4.09 | | **<.001** | |
| Middle temporal gyrus | L | | 231 | | -57 | -68 | 15 | 3.97 | | **<.001** | |
| Middle frontal gyrus, superior frontal gyrus | R | | 447 | | 36 | 44 | 32 | 3.80 | | **<.001** | |
| Thalamus | L/R | | 770 | | 4 | -6 | 4 | 3.79 | | **<.001** | |
| Insula, rolandic operculum | R | | 201 | | 44 | 0 | 2 | 3.77 | | **<.001** | |
| Cerebelum | R | | 244 | | 33 | -74 | -50 | 3.48 | | **<.001** | |
| *HC<MDD* | - | | - | | - | - | - | - | | n.s. | |
| *CON>MDD* |  | |  | |  |  |  |  | |  | |
| Inferior temporal gyrus, fusiform gyrus | L | | 218 | | -52 | -51 | -20 | 3.83 | | **<.001** | |
| Middle cingulate & paracingulate gyri | L/R | | 301 | | -4 | -21 | 44 | 3.75 | | **<.001** | |
| Middle temporal gyrus, inferior temporal gyrus, middle occipital gyrus, inferior occipital gyrus | L | | 253 | | -50 | -68 | 9 | 3.60 | | **<.001** | |
| *CON<MDD* | - | | - | | - | - | - | - | | n.s. | |
| *HC>CON* | - | | - | | - | - | - | - | | n.s. | |
| *HC<CON* | - | | - | | - | - | - | - | | n.s. | |
|  | Remission sample^2^ (*n*=1310) | | | | | | | | | | |
| *Main effect group* | - | | - | | - | - | - | - | | n.s. | |
| *HC>MDD* | - | | - | | - | - | - | - | | n.s. | |
| *HC<MDD* | - | | - | | - | - | - | - | | n.s. | |
| *CON>MDD* | - | | - | | - | - | - | - | | n.s. | |
| *CON<MDD* | - | | - | | - | - | - | - | | n.s. | |
| *HC>CON* | - | | - | | - | - | - | - | | n.s. | |
| *HC<CON* | - | | - | | - | - | - | - | | n.s. | |
|  | Recurrence sample^3^ (*n*=1109) | | | | | | | | | | |
| *Main effect group* | - | | - | | - | - | - | - | | n.s. | |
| *HC>MDD* | - | | - | | - | - | - | - | | n.s. | |
| *HC<MDD* | - | | - | | - | - | - | - | | n.s. | |
| *CON>MDD* | - | | - | | - | - | - | - | | n.s. | |
| *CON<MDD* | - | | - | | - | - | - | - | | n.s. | |
| *HC>CON* | - | | - | | - | - | - | - | | n.s. | |
| *HC<CON* | - | | - | | - | - | - | - | | n.s. | |

*Note.* HC=healthy controls, CON=converters, MDD=major depressive disorder.

^1^ only significant clusters (*p_unc_*-value < .001) with cluster size *k*≥200 are reported.

^2^ Includes remitted MDD patients only.

^3^ Includes remitted MDD patients only who experienced a relapse within 2-year follow-up. Matched for sex, age, scanner variables.

### Supplementary Table 7

*Results from ANCOVAs and t-tests in the smaller sample matched for age, sex and scanner variables for the amygdala (n=135)*

|  |  |  | Peak voxel coordinates | | |  |  |  |
| --- | --- | --- | --- | --- | --- | --- | --- | --- |
| Contrast | Side | Cluster size | x | y | z | *F-/t-*value | *p_tfce-FWE_*-value | Effect size partial  η²/*d* |
| Main effect group | R | 60 | 32 | 0 | -27 | 4.10 | **.042** | .060 |
| HC>MDD | - | - | - | - | - | - | .371 | **-** |
| HC<MDD | - | - | - | - | - | - | .373 | - |
| CON>MDD | R | 226 | 21 | 6 | -18 | 2.78 | **.016** | .490 |
| CON<MDD | - | - | - | - | - | - | >.999 | - |
| HC>CON | - | - | - | - | - | - | >.999 | - |
| HC<CON | - | - | - | - | - | - | .080 | - |

*Note.* HC=healthy controls, CON=converters, MDD=major depressive disorder. Significant *p*-values of the significance level *p* < .05 are highlighted in bold.

### Supplementary Table 8

*Results from exploratory whole-brain analyses in the smaller sample matched for age, sex and scanner variables (n=135) conducted at p<.001, uncorrected, with a cluster threshold of k=200*

|  |  |  | | Peak voxel coordinates | | | | |  | |  |
| --- | --- | --- | --- | --- | --- | --- | --- | --- | --- | --- | --- |
| Anatomical region | Side | | Cluster size^1^ | | x | y | z | *t-/F-*value | | *p_unc_*-value | |
| *Main effect group* |  | |  | |  |  |  |  | |  | |
| Middle temporal gyrus, middle occipital gyrus | L | | 424 | | -48 | -68 | 10 | 12.50 | | **<.001** | |
| Middle temporal gyrus | L | | 249 | | -60 | -22 | -12 | 3.79 | | **<.001** | |
| Middle temporal gyrus, superior temporal gyrus | R | | 398 | | 62 | -6 | -15 | 3.68 | | **<.001** | |
| *HC>MDD* |  | |  | |  |  |  |  | |  | |
| Postcentral gyrus, superior parietal gyrus | R | | 308 | | 40 | -39 | 64 | 4.61 | | **<.001** | |
| *HC<MDD* | - | | - | | - | - | - | - | | n.s. | |
| *CON>MDD* |  | |  | |  |  |  |  | |  | |
| Middle temporal gyrus, middle occipital gyrus | L | | 967 | | -48 | -66 | 9 | 4.97 | | **<.001** | |
| Inferior frontal gyrus, opercular part, inferior frontal gyrus, triangular part | L | | 511 | | -56 | 20 | 0 | 4.56 | | **<.001** | |
| Middle temporal gyrus, superior temporal gyrus | R | | 1002 | | 62 | -6 | -14 | 4.37 | | **<.001** | |
| Middle temporal gyrus, inferior temporal gyrus | L | | 818 | | -58 | -27 | -12 | 4.36 | | **<.001** | |
| Middle temporal gyrus, inferior temporal gyrus | L | | 548 | | -51 | -48 | -3 | 4.06 | | **<.001** | |
| Middle cingulate & paracingulate gyri | L | | 233 | | -6 | -28 | 42 | 3.77 | | **<.001** | |
| *CON<MDD* | - | | - | | - | - | - | - | | n.s. | |
| *HC>CON* | - | | - | | - | - | - | - | | n.s. | |
| *HC<CON* |  | |  | |  |  |  |  | |  | |
| Middle cingulate & paracingulate gyri, precuneus, paracentral lobule | L | | 209 | | -10 | -36 | 54 | 3.99 | | **<.001** | |
| Middle temporal gyrus | R | | 205 | | 64 | -8 | -18 | 3.61 | | **<.001** | |

*Note.* HC=healthy controls, CON=converters, MDD=major depressive disorder. Includes remitted MDD patients only who experienced a relapse within 2-year follow-up. Significant *p*-values of the significance level *p* < .05 are highlighted in bold.

### Supplementary Table 9

*Results from additional analyses in the total sample with psychotropic medication, familial risk for MDD, and education level as additional covariates for the dorsolateral prefrontal cortex, insula and amygdala*

|  |  |  | Peak voxel coordinates | | |  |  |  |
| --- | --- | --- | --- | --- | --- | --- | --- | --- |
| Contrast | Side | Cluster size^1^ | x | y | z | *t-/F-*value | *p_tfce-FWE_*-value | Effect size partial  η²/*d* |
|  | Psychiatric medication | | | | | | | |
| *DLPFC* |  |  |  |  |  |  |  |  |
| Main effect group | - | - | - | - | - | - | >.999 | - |
| HC>MDD | - | - | - | - | - | - | .352 | - |
| HC<MDD | - | - | - | - | - | - | .493 | - |
| CON>MDD | R | 346 | 34 | 42 | 30 | 2.42 | **.013** | .371 |
| CON<MDD | - | - | - | - | - | - | .065 | - |
| HC>CON | - | - | - | - | - | - | .194 | - |
| HC<CON | - | - | - | - | - | - | .93 | - |
| *Insula* |  |  |  |  |  |  |  |  |
| Main effect group | - | - | - | - | - | - | .170 | - |
| HC>MDD | - | - | - | - | - | - | .106 | - |
| HC<MDD | - | - | - | - | - | - | .528 | - |
| CON>MDD | R | 1422 | 46 | 14 | -9 | 3.13 | **<.001** | .480 |
|  | L | 1308 | -48 | 3 | 4 | 1.91 | **.004** | .293 |
| CON<MDD | - | - | - | - | - | - | .063 | - |
| HC>CON | - | - | - | - | - | - | .224 | - |
| HC<CON | - | - | - | - | - | - | .272 | - |
| *Amygdala* |  |  |  |  |  |  |  |  |
| Main effect group | R | 121 | 30 | -3 | -24 | 6.70 | **.027** | .008 |
| HC>MDD | - | - | - | - | - | - | .078 | - |
| HC<MDD | - | - | - | - | - | - | >.999 | - |
| CON>MDD | R | 526 | 30 | -3 | -24 | 3.54 | **<.001** | .543 |
|  | L | 276 | -28 | -4 | -24 | 1.71 | **.014** | .262 |
| CON<MDD | - | - | - | - | - | - | .209 | - |
| HC>CON | - | - | - | - | - | - | .264 | - |
| HC<CON | R | 181 | 30 | -3 | -24 | 2.93 | **.017** | .447 |
|  | Familial risk for MDD | | | | | | | |
| *DLPFC* |  |  |  |  |  |  |  |  |
| Main effect group | R | 122 | 34 | 44 | 30 | 9.57 | **.028** | .011 |
| HC>MDD | R | 1008 | 38 | 44 | 32 | 3.91 | **.004** | .193 |
| HC<MDD | - | - | - | - | - | - | .812 | - |
| CON>MDD | - | - | - | - | - | - | .327 | - |
| CON<MDD | - | - | - | - | - | - | .159 | - |
| HC>CON | - | - | - | - | - | - | .484 | - |
| HC<CON | - | - | - | - | - | - | .397 | - |
| *Insula* |  |  |  |  |  |  |  | - |
| Main effect group | R | 52 | 44 | 0 | 3 | 7.22 | **.044** | .008 |
|  | R | 8 | 46 | 14 | -9 | 7.11 | **.049** | .008 |
| HC>MDD | R | 937 | 44 | 0 | 2 | 3.75 | **.007** | .185 |
| HC<MDD | - | - | - | - | - | - | .828 | - |
| CON>MDD | - | - | - | - | - | - | .053 | - |
| CON<MDD | - | - | - | - | - | - | .140 | - |
| HC>CON | - | - | - | - | - | - | .591 | - |
| HC<CON | R | 70 | 46 | 15 | -9 | 2.71 | .**026** | .414 |
| *Amygdala* |  |  |  |  |  |  |  |  |
| Main effect group | R | 68 | 30 | -3 | 24 | 5.73 | **.040** | .007 |
| HC>MDD | L | 47 | -20 | 0 | 16 | 2.87 | **.039** | .141 |
| HC<MDD | - | - | - | - | - | - | >.999 | - |
| CON>MDD | R | 326 | 30 | -3 | -24 | 3.33 | **.002** | .511 |
| CON<MDD | - | - | - | - | - | - | .359 | - |
| HC>CON | - | - | - | - | - | - | .790 | - |
| HC<CON | R | 373 | 30 | -3 | 24 | 2.97 | **<.001** | .453 |
|  | Education level | | | | | | | |
| *DLPFC* |  |  |  |  |  |  |  |  |
| Main effect group | - | - | - | - | - | - | .083 | - |
| HC>MDD | R | 435 | 36 | 42 | 32 | 3.48 | **.023** | .170 |
| HC<MDD | - | - | - | - | - | - | .632 | - |
| CON>MDD | - | - | - | - | - | - | .081 | - |
| CON<MDD | - | - | - | - | - | - | .637 | - |
| HC>CON | L | 1 | -32 | 26 | 56 | 2.84 | **.050** | .139 |
| HC<CON | - | - | - | - | - | - | >.999 | - |
| *Insula* |  |  |  |  |  |  |  |  |
| Main effect group | - | - | - | - | - | - | .079 | - |
| HC>MDD | R | 384 | 44 | 0 | 2 | 3.58 | **.019** | .175 |
| HC<MDD | - | - | - | - | - | - | .614 | - |
| CON>MDD | R | 623 | 46 | 14 | -10 | 3.07 | **.006** | .150 |
| CON<MDD | - | - | - | - | - | - | .787 | - |
| HC>CON | - | - | - | - | - | - | .060 | - |
| HC<CON | - | - | - | - | - | - | .463 | - |
| *Amygdala* |  |  |  |  |  |  |  |  |
| Main effect group | R | 92 | 30 | -3 | 24 | 6.62 | **.029** | .008 |
| HC>MDD | - | - | - | - | - | - | .065 | - |
| HC<MDD | - | - | - | - | - | - | >.999 | - |
| CON>MDD | R | 475 | 30 | -3 | 24 | 3.62 | **<.001** | .177 |
|  | L | 32 | -28 | -4 | -24 | 1.85 | **.035** | .091 |
| CON<MDD | - | - | - | - | - | - | .999 | - |
| HC>CON | - | - | - | - | - | - | .107 | - |
| HC<CON | R | 164 | 30 | -3 | 24 | 3.30 | **.014** | .162 |

*Note.* HC = healthy controls, CON = converters, MDD = major depressive disorder. Significant *p*-values of the significance level *p* < .05 are highlighted in bold.

### Supplementary Table 10

*Kolmogorov-Smirnov test results for normality of gray matter volumes across regions of interest*

| Region of interest | D | *p*-value |
| --- | --- | --- |
| Hippocampus | 0.02 | .307 |
| Amygdala | 0.02 | .273 |
| DLPFC | 0.05 | **<.001** |
| rACC | 0.03 | **.045** |
| Insula | 0.04 | **.007** |

*Note.* DLPFC=dorsolateral prefrontal cortex, rACC= rostral anterior cingulate cortex. Significant *p*-values of the significance level *p* < .05 are highlighted in bold.

### Supplementary Figure 1
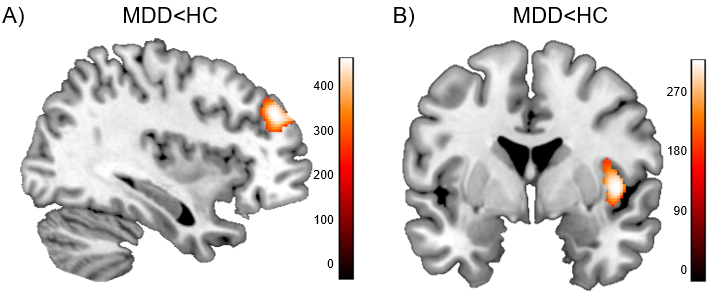


Gray matter volume reductions in the total sample of patients with MDD vs. healthy controls (MDD<HC *t*-contrast) significant at *p*_tfce-FWE_<.05. Depicted are the significant clusters **A)** within the dorsolateral prefrontal cortex ROI at x=222, y=303, z=204 and **B)** within the insula ROI at x=231, y=215, z=144. Color bar: TFCE values.

###
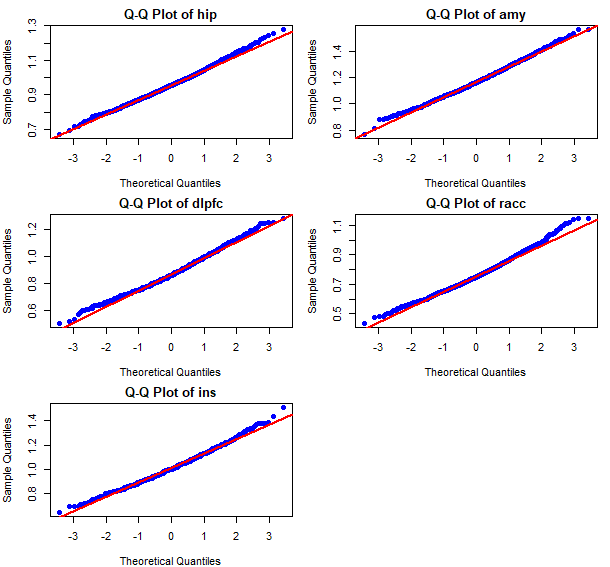
Supplementary Figure 2

Q-Q plots of residuals for each region of interest, including the hippocampus (hip), amygdala (amy), dorsolateral prefrontal cortex (dlpfc), anterior cingulate cortex (acc), and insula (ins). Each Q-Q plot compares the observed quantiles of the residuals to the expected quantiles of a normal distribution. Points lying along the diagonal reference line suggest that the data are approximately normally distributed, while deviations from the line indicate departures from normality.

###
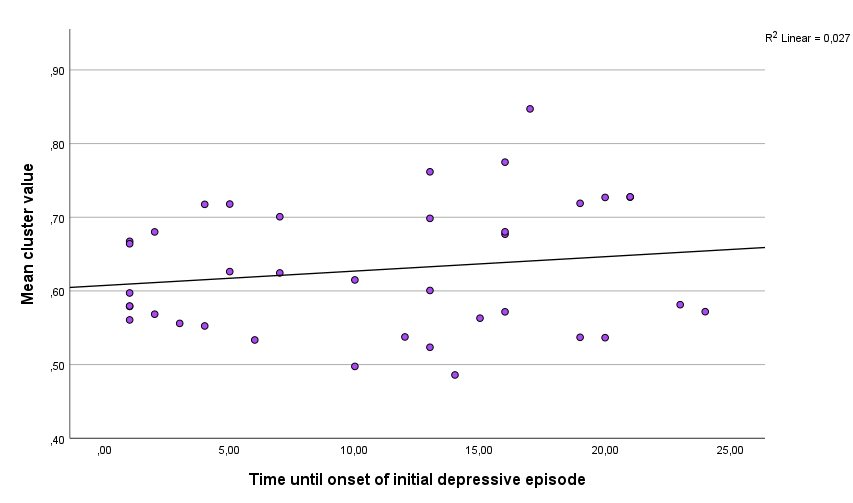
Supplementary Figure 3

Relation of amygdala gray matter volumes and time until onset of the initial depressive episode in converters (*n*=38). No significant association of mean cluster values of the significant amygdala cluster derived from the F-test in analysis 1 (x=30, y=-3, z=-24) with time until onset of the initial depressive episode in the converter subsample (*r*=.17, *p*=.32). For *n*=7 converters information on exact time of onset of the first depressive episode was missing. GMV=gray matter volumes.

###
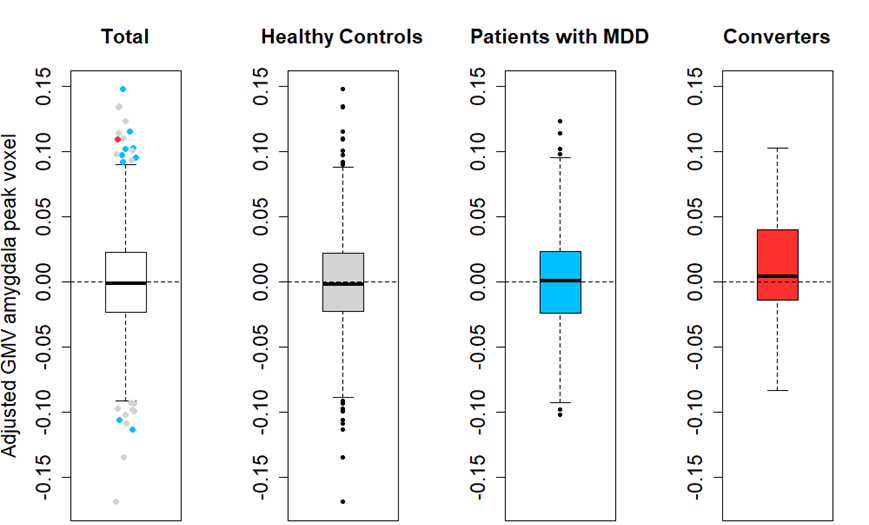
Supplementary Figure 4

Boxplots of the residuals from the linear model, adjusting the GMV from the amygdala peak voxel derived from the significant cluster identified in analysis 1 (x = 30, y = -3, z = -24). MDD=major depressive disorder.

###
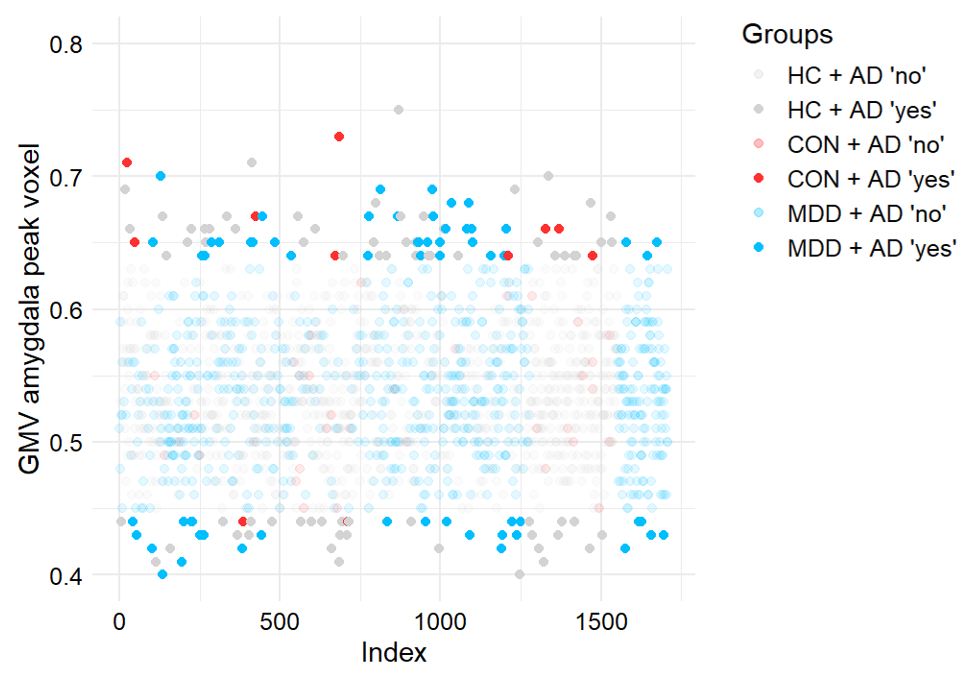
Supplementary Figure 5

Results of the isolation forest approach applied to the gray matter values of the significant amygdala cluster of analysis 1, extracted at the peak voxel: x = 30, y = -3, z = -24. The plot shows the values of individuals categorized by group (healthy controls (HC), *n*=916, gray; converters (CON), *n*=45, red; patients with major depressive disorder (MDD), *n*=748, blue) and anomaly detection (AD) status (non-anomalous = low opacity, anomalous = high opacity).

## References

1. Kircher T, Wöhr M, Nenadic I, Schwarting R, Schratt G, Alferink J, et al. Neurobiology of the major psychoses: a translational perspective on brain structure and function—the FOR2107 consortium. Eur Arch Psychiatry Clin Neurosci. 2019;269:949–962.

2. Wittchen HU, Wunderlich U, Grushwitz S, Zaudig M. SKID I. Strukturiertes Klinisches Interview für DSM-IV. Achse I: Psychische Störungen. Interviewheft und Beurteilungsheft. Eine Deutschsprachige, Erweiterte Bearbeitung Der Amerikanischen Originalversion Des SCID I. 1997. 1997.

3. Hassel S, Almeida JR, Kerr N, Nau S, Ladouceur CD, Fissell K, et al. Elevated striatal and decreased dorsolateral prefrontal cortical activity in response to emotional stimuli in euthymic bipolar disorder: no associations with psychotropic medication load. Bipolar Disorders. 2008;10:916–927.

4. Reynolds CR, Fletcher‐Janzen E, editors. Encyclopedia of Special Education. 1st ed. Wiley; 2008.

5. Vogelbacher C, Möbius TWD, Sommer J, Schuster V, Dannlowski U, Kircher T, et al. The Marburg-Münster Affective Disorders Cohort Study (MACS): A quality assurance protocol for MR neuroimaging data. NeuroImage. 2018;172:450–460.

6. Flinkenflügel K, Meinert S, Thiel K, Winter A, Goltermann J, Strathausen L, et al. Negative Stressful Life Events and Social Support Are Associated With White Matter Integrity in Depressed Patients and Healthy Control Participants: A Diffusion Tensor Imaging Study. Biological Psychiatry. 2023;94:650–660.

7. Lalousis PA, Schmaal L, Wood SJ, Reniers RLEP, Barnes NM, Chisholm K, et al. Neurobiologically Based Stratification of Recent-Onset Depression and Psychosis: Identification of Two Distinct Transdiagnostic Phenotypes. Biological Psychiatry. 2022;92:552–562.

8. Radua J, Vieta E, Shinohara R, Kochunov P, Quidé Y, Green MJ, et al. Increased power by harmonizing structural MRI site differences with the ComBat batch adjustment method in ENIGMA. NeuroImage. 2020;218:116956.

9. Fortin J-P, Parker D, Tunç B, Watanabe T, Elliott MA, Ruparel K, et al. Harmonization of multi-site diffusion tensor imaging data. NeuroImage. 2017;161:149–170.

10. Fortin J-P, Cullen N, Sheline YI, Taylor WD, Aselcioglu I, Cook PA, et al. Harmonization of cortical thickness measurements across scanners and sites. NeuroImage. 2018;167:104–120.

11. Cortes D. isotree: Isolation-Based Outlier Detection. 2019:0.6.1-1.
